# The effectiveness of video animations as information tools for patients and the general public: A systematic review

**DOI:** 10.3389/fdgth.2022.1010779

**Published:** 2022-10-31

**Authors:** Thirimon Moe-Byrne, Ella Evans, Nadia Benhebil, Peter Knapp

**Affiliations:** ^1^ Department of Health Sciences, University of York, York, United Kingdom; ^2^ Hull York Medical School, University of York, York, United Kingdom

**Keywords:** video animations, information tools, patients, knowledge, attitudes and cognition, behaviors

## Abstract

**Background and objectives:**

Video animations are used increasingly as patient information tools; however, we do not know their value compared to other formats of delivery, such as printed materials, verbal consultations or static images.

**Methods:**

This review compares the effectiveness of video animations as information tools vs. other formats of delivery on patient knowledge, attitudes and cognitions, and behaviours. Included studies had the following features: controlled design with random or quasi-random allocation; patients being informed about any health condition or members of the public being informed about a public health topic; comparing video animation with another delivery format. Multiple digital databases were searched from 1996-June 2021. We also undertook citation searching. We used dual, independent decision-making for inclusion assessment, data extraction and quality appraisal. Included studies were appraised using the Cochrane ROB2 tool. Findings were reported using narrative synthesis.

**Results:**

We included 38 trials, focussed on: explaining medical or surgical procedures (*n* = 17); management of long-term conditions (*n* = 11); public health, health-promotion or illness-prevention (*n* = 10). Studies evaluated cartoon animations (*n* = 29), 3D animations (*n* = 6), or 2D animations, “white-board” animations or avatars (*n* = 1 each). Knowledge was assessed in 30 studies, showing greater knowledge from animations in 19 studies, compared to a range of comparators. Attitudes and cognitions were assessed in 21 studies, and animations resulted in positive outcomes in six studies, null effects in 14 studies, and less positive outcomes than standard care in one study. Patient behaviours were assessed in nine studies, with animations resulting in positive outcomes in four and null effects in the remainder. Overall risk of bias was “high” (*n* = 18), “some concerns” (*n* = 16) or “low” (*n* = 4). Common reasons for increased risk of bias were randomisation processes, small sample size or lack of sample size calculation, missing outcome data, and lack of protocol publication.

**Discussion:**

The overall evidence base is highly variable, with mostly small trials. Video animations show promise as patient information tools, particularly for effects on knowledge, but further evaluation is needed in higher quality studies.

**Systematic Review Registration:**

https://www.crd.york.ac.uk/prospero/display_record.php?, identifier: CRD42021236296.

## 1. Background

Easy access to the internet has made online and digital health communication both possible and attractive, and many people consider the internet a valuable tool for finding health information (1–3). This in turn has generated opportunities for the use of multimedia in delivering health information to patients, which may provide benefits both for patients and healthcare providers.

Traditionally, information has been provided to patients through face-to-face clinical consultation, information leaflet (with or without images) or, in the case of some public health issues, short TV film. However, patients do not always understand what is being explained to them, perhaps due to cultural and educational gaps between clinicians and patients (4). This problem is especially relevant for people with low health literacy. This group of people find some health-related information difficult to understand and research suggests that a “high information burden” could actually discourage them from taking part in assessments of their health, such as screening (5).

The application of new technologies to patient communication has provided alternative methods for bringing information to patients and their families, with potential advantages. For example, the Scientific Animations Without Borders (SAWBO) organisation has generated dozens of short animation films, available in multiple languages, on a range of public health topics (6). There is growing evidence for the benefits of multimedia information in enhancing patients' satisfaction with information and improving knowledge retention (4, 7). Use of multimedia, such as video, animations and static images in delivering health information can help patients understand their condition better than words alone. There is evidence that graphics and animations enhance knowledge and the recall of facts related to specific healthcare interventions (8, 9).

Research using non-randomisedstudy designs has shown that animations are better at communicating a complex biological process to patients than a graphic with a figure legend (10), and they can be more effective than static sequential images for teaching dynamic events (11, 12). Animations can also highlight important content better than a photographic video (13), and edited animations may be more acceptable to patients who do not want to see realistic portrayals of medical interventions (13, 14). Animations can also help to overcome language barriers and educate patients with limited literacy skills (15). Outside healthcare settings a meta-analysis of controlled experiments found that animations improved learning (either knowledge or procedural skills) more than static images (16). However, there are also concerns that animations may encourage only surface learning, and that patients' attention to animations may be time-limited.

Considering the potential advantages and disadvantages of animations as patient information tools, we do not know their comparative value against other forms of information delivery in healthcare. This systematic review seeks to address this gap by evaluating the effectiveness of video animations as information tools on patients' knowledge, attitudes and cognitions, and behaviours.

## 2. Methods

The review protocol was registered with PROSPERO in February 2021 under ID CRD42020084714 (Available from: https://www.crd.york.ac.uk/prospero/display_record.php?ID=CRD42021236296).

### 2.1. Eligibility criteria

Participants were either patients in a healthcare setting or members of the public being informed about a topic relevant to public health, health promotion or illness prevention. Studies were eligible for inclusion if they used a randomised or quasi-randomised controlled design, and compared a video animation (e.g., cartoons, avatars, “white board” animation, or animated 2D or 3D models) with another format of information delivery (e.g., print, audio recording, “talking head” video, video of a procedure, spoken information) either as an alternative or additional format. Video animations of any length, with or without voiceover were eligible. Animations were eligible if they were part of a multi-component information package as long as the effect of the animation could be isolated. We excluded studies if: they did not include a control arm; or reported a hypothetical scenario; or the animation was compared with no information intervention.

The primary outcome was knowledge, and secondary outcomes were attitudes and cognitions (that is, feelings or thoughts, such as satisfaction with information, self-confidence) and behaviours (that is, actions or intended actions, such as condition self-management skills, appointment attendance, or behavioural intentions).

### 2.2. Search strategy

Relevant studies were identified by searching the following electronic databases: MEDLINE, EMBASE, Cumulative Index to Nursing and Allied Health Literature (CINAHL), PsycINFO and the Cochrane Central Register of Controlled Trials (CENTRAL) published from January 1996 onwards. Additional searches were undertaken on Open Grey (opengrey.eu). Forwards- and backwards-citation searching was also undertaken through Google Scholar and the reference lists of all included articles (See Supplementary S1: Full search strategy). We did not apply any language restrictions. The searches were conducted in June 2021 and were supported by a specialist information scientist from the Centre for Reviews and Dissemination (CRD) at the University of York.

### 2.3. Study selection

**Figure 1:**
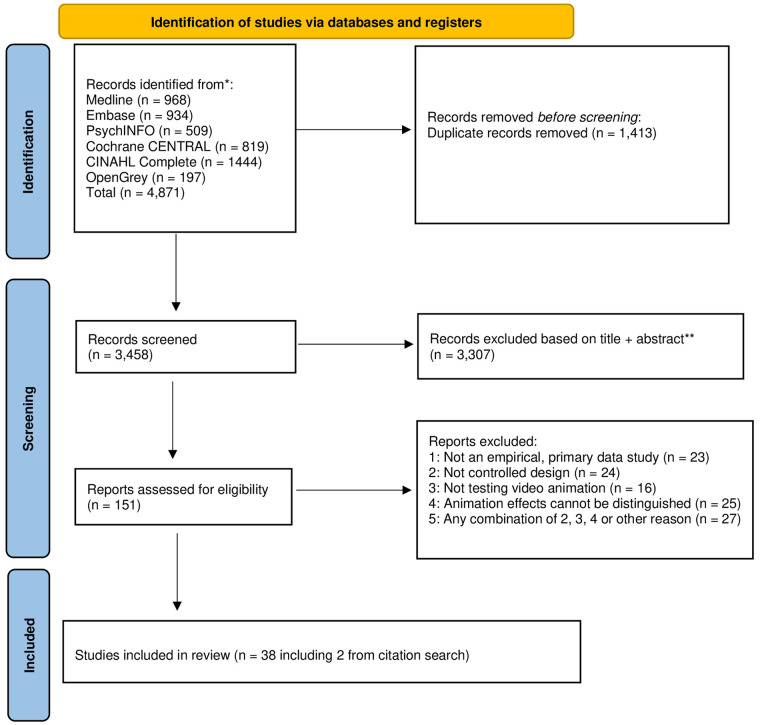
PRISMA flow diagram.

The studies retrieved from the searches were exported into EndNote and de-duplicated. Two reviewers (EE, NB) independently screened titles and abstracts of all records identified in the search using pre-defined criteria, and then by full text article. Disagreements were resolved through consensus or by consultation with a third reviewer (PK) (See Figure 1 for PRISMA flowchart).

### 2.4. Data extraction

We used a piloted data extraction form to extract the following data: basic study information; details of participants; type of intervention and control arms(s); details of the intervention; outcome data. Data extraction was conducted by one reviewer (NB, TMB or PK) and checked by a second reviewer. Any disagreements were resolved through consensus, and if necessary, a third reviewer was consulted.

### 2.5. Quality assessment

We used the Cochrane Risk of Bias Tool-2 (RoB 2) (17) to assess the methodological quality of all included trials on the following five criteria: randomisation process; deviation from intended interventions; missing outcome data; outcome measurement; and selection of the reported result. Risk of bias assessment was conducted by one reviewer (NB, TMB or PK) and checked by a second reviewer. Discrepancies were resolved by discussion, with involvement of a third reviewer if necessary.

### 2.6. Data synthesis

Due to the degree of heterogeneity among the included trials, formal meta-analysis was not feasible. Therefore, a narrative approach was used, summarising the findings according to three pre-identified outcome categories (knowledge; attitudes and cognitions; behaviours) and *de novo* the intended purpose of the interventions. We have taken reports of differences between trials arms of *p* < .05 as indicators of effect.

## 3. Results

### 3.1. Study characteristics

The database searches produced 3,458 unique hits (see Figure 1). Sifting by title and abstract and then by full text, resulted in 36 eligible studies. A further two studies were added following citation searches, producing a total of 38 included studies.

The 38 studies comprised 35 trials using random allocation and 3 using quasi-random methods. All but two of the trials used individual participant allocation; in two trials allocation had been clustered.

Study samples ranged from 30 to 843 participants (median 101); the included trials recruited a total of 5,646 participants. There was a wide spread of geographical locations, with the studies being undertaken in 19 different countries: eight from the USA; four from Australia; three each from Thailand and New Zealand; two each from Austria, Indonesia, Korea, Netherlands and the United Kingdom; and one each from Belgium, Canada, China, France, India, Iran, Japan, Tanzania, Turkey and Singapore. All but two of the studies were reported in English language journals; the exceptions were studies reported in German (18) and Korean (19). (See Tables 1–3 for summary of study features).

**Table 1:** Category 1 findings (explaining medical or surgical procedures).

Authors, years	study design	ROB	Participants	sample size	Knowledge	Attitude	Behaviour
Lattuca, 2018	RCT	Low	Patients undergoing coronary angiography	843	↑	↑	–
Mhalu, 2015	RCT	Low	Patients at risk of pulmonary tuberculosis	200	–	–	↑
Winter, 2016	RCT	Low	Patients with acute renal colic	92	↑	↔	–
Miao, 2020	RCT	Some	Patients referred for Mohs micrographic surgery	102	↑	↔	–
Reynolds-Wright, 2020	RCT and Quasi RCT	Some	Gynaecological patients with confirmed gestation	172	↖	↖	–
Sahebalam, 2020	RCT	Some	Primary school children referred for dental surgery	50	–	–	↑
Sari Turk, 2020	RCT	Some	Patients awaiting stem cell transplantation	82	–	↖	–
Tipotsch, 2016	RCT	Some	Patients awaiting cataract surgery	123	↑	↔	–
Hermann, 2002	RCT	High	Patients undergoing thyroid surgery	80	↔	↖	–
Hong, 2012	RCT	High	Patients about to undergo CT scan	150	↑	↑	–
Kakinuma, 2011	RCT	High	Patients about to undergo surgery for cancer	211	↑	–	–
Mayilvaganan, 2018	RCT	High	Patients undergoing thyroid surgery	60	–	↖	–
Mednick, 2016	RCT	High	Patients undergoing an initial IVFA investigation	52	↑	↔	–
Mladenovski, 2008	RCT	High	Patients referred for dental surgery	30	↔	↖	–
Platto, 2019	RCT	High	Patients awaiting skin surgery	45	–	↔	–
Tou, 2013	RCT	High	Patients undergoing bowel surgery	31	↔	↔	–
Yap, 2019	RCT (3:1)	High	Patients undergoing coronary angiography	332	↑	–	–

↑ Favours animation; ↖ Some positive results with animation; ↔ No difference between groups; –, not assessed; ROB, Risk of bias; Some, Some risk of bias concern, IVFA, Intravenous fluorescein angiography.

**Table 2:** Category 2 findings (management of long-term conditions).

Authors, years	Study design	ROB	Participants	Sample size	Knowledge	Attitude	Behaviour
Baker, 2018	RCT	Some	Patients undergoing testing for chronic constipation	100	↔	-	-
Chakravarthy, 2018	RCT	Some	Patients prescribed opioids in Emergency Departments	52	↑	–	–
Cleeran, 2014	RCT	Some	Patients with periodontitis	67	↑	–	–
Jones, 2019	RCT	Some	Patients after surgery	96	–	↖	–
Kayler, 2020	RCT	Some	Kidney donation, patients	80	↑	↔	↖
Li, 2019	RCT	Some	Patients with lung cancer, preparing for surgery	80	↑	–	↔
Mofrad Babapour, 2021	RCT	Some	Patients attending a memory clinic	203	↑	↔	–
Caldero, 2014	RCT	High	Latino/Hispanic patients with Type 2 Diabetes	240	↖	–	–
Jones, 2016	RCT	High	Patients with acute coronary syndrome	70	↖	↖	↖
Saengrow, 2018	RCT	High	Use of anti-epileptics, paediatric patients	214	↑	–	↑
Wonggom, 2020	RCT	High	Patients with heart failure	36	↖	↔	↔

↑ Favours animation; ↖ Some positive results with animation; ↔ No difference between groups; ED, Emergency department; ROB, Risk of bias; Some, Some risk of bias concerns;.

**Table 3:** Category 3 finding (topics related to public health, health promotion or illness prevention).

Authors, years	Study design	ROB	Participants	Sample size	Knowledge	Attitude	Behaviour
Burapasikarin, 2020	RCT	Low	Postpartum women	270	–	–	↑
Bukkhunthod, 2020	Cluster RCT	Some	School children aged 9 to 12 years	80	↑	–	–
Choa, 2008	Cluster RCT	Some	Hospital employees	85	–	–	↖
Housten, 2020	RCT	Some	People using a community food bank or attending the Houston Cancer Prevention Centre	187	↔	–	–
Meppelink, 2015	RCT	Some	Participants 55 + with either low or high health literacy	231	↖	–	–
Leiner, 2004	RCT	High	Parents of children receiving polio vaccines	192	↑	–	–
Rakhmilla, 2018	Quasi RCT	High	Senior High School students	180	↑	–	–
Romantika, 2020	Quasi RCT	High	Mothers of children aged 4–7 years	120	↑	↑	–
Ruparel, 2019	RCT	High	Smokers/former smokers	246	↑	↑	–
Schnellinger, 2010	RCT	High	Parents of paediatric patients	162	↖	↔	–

↑ Favours animation; ↖ Some positive results with animation; ↔ No difference between groups; ROB, Risk of bias; Some, Some risk of bias concerns.

#### 3.1.1. Topic and style of the animations

Cartoon animations were used in 29 studies, on the subject of: kidney donation (20); post-surgery rehabilitation (21); contraception (22); Type 2 diabetes (23); use of opioids (24); acute coronary syndrome and maintaining heart health (25); polio vaccination (9); thyroid surgery (26) in one arm; chronic constipation (27); liver fluke (28); cardio-pulmonary resuscitation (CPR) (29); consent to CT scanning (19); colorectal cancer screening (30, 31); preparation for preparation for surgery (13, 32), or skin surgery (33, 34), or angiography (35); sputum testing (36); prevention of thalassemia (37); early medical abortion (38); lung cancer screening (39); medicines for epilepsy (40); preventive dental care (41); stem cell transplantation (42); appropriate antibiotic use (8); consent for cystoscopy (43); lumbar puncture (44).

3D animated models were used in six studies to portray: periodontitis (45); maintaining post-operative health (18, 46); dental extraction (47); angiography (48); and cataract surgery (49).

2D animated video was used in one trial with mothers of children with behavioural problems (50).

A “white board” animation was used in one trial to explain angiography (51), and an avatar was used in one trial to explain living well with heart failure (52).

The duration of the animations ranged from 1.25 to 31 min, although in three study reports the animation duration was not stated (29, 49, 52).

Thirteen of the 38 articles (34%) included a link to the tested animation; in 25 articles no link was provided.

#### 3.1.2. Comparators and alternatives to animations

In 14 trials the animation was provided in addition to control group interventions, which were:

• standard care (22, 25, 44, 46, 48, 52);
• consultation with surgeon, anaesthetist or other doctor (i.e. spoken information) (13, 34, 40);
• booklet (32, 39);
• standard written and spoken information (42, 49);
• nurse education audio-recording (20).

In 23 trials the animation was provided as an alternative to control group interventions, which were:

• spoken information (19, 21, 33);
• standard care (35, 36, 38);
• easy-to-read written information (24, 53);
• static images (31, 45);
• either diagram or 3D model, according to allocation (26);
• written booklet (8, 18, 27, 47, 48); printed information (9, 50); booklets, posters and spoken information (28);
• live instructions provided by phone (29);
• audio-booklet or static images, according to allocation (30);
• peer education or conventional lecture, according to allocation (37);
• Tell-Show-Do technique (41);
• verbal consent following provision of spoken information (43).

In one trial (51) the animation was provided as an alternative to the standard physician-patient consent conversation in one trial arm, and in addition to it in another trial arm.

#### 3.1.3. Access to animations

The level of access that participants had to the animations was stated in 22 of the 38 trial reports. In 14 studies they viewed the animation only once (8, 18–20, 22, 27, 30, 36, 38, 40–42, 48, 50) and in one study only once or twice as they preferred (39). In two studies they viewed the animation exactly twice (21) or three times (28). In four studies animation viewing was unlimited (25, 52) or unlimited during the clinic visit (45). In one 3-arm trial, patients were allowed to watch it only once if they were in the clinic (clinic viewing arm) or had unlimited viewing if they were at home (home viewing arm) (44).

In 16 studies level of access was not stated (9, 13, 23, 24, 29, 31–35, 37, 43, 46, 47, 49, 51).

#### 3.1.4. Outcome measures

Knowledge was the most commonly reported outcome in 30 trials (8, 9, 13, 18–21, 23–25, 27, 28, 30–33, 35, 37–40, 43–45, 47–52).

Attitudes and cognitions were reported in 20 trials, reporting self-efficacy (20); information satisfaction (18, 32, 33, 38, 42–44, 47–49, 51); illness perceptions (25); perceptions of surgery, quality of recovery (46); information satisfaction, unmet information needs (26); information satisfaction, familiarity with topic (19); desire for information (34); self-care confidence (52); attitude to information (50); subjective knowledge, decisional certainty (39); information satisfaction, having learned from information (8).

Nine trials reported behaviour outcomes, including willingness to give consent and undergo the procedure (20); physical activity (21); contraception use (22); return to work, physical activity and medication adherence (25); CPR skills, time taken to initiate CPR (29); self-care behaviours (52); quality of sputum sample (36); medication adherence (40); patient co-operation (41).

Only two trials reported all three categories of outcome (25, 52).

#### 3.1.5. Timing of outcome assessment

In 35 studies outcomes were assessed shortly after delivery of the information intervention. However, in five of these studies there was an additional assessment of outcomes (at the second dental appointment (41); 1 day later (32); 2 weeks later (45); 7 weeks later (25); 3 months later (40); or 4 weeks later (8)).

In two studies outcome assessment was only made some time after intervention delivery (30 and 90 days later (52); 6–8 weeks later (22)), and in one study (29) outcomes were assessed at the same time as participants were receiving the intervention.

### 3.2. Outcomes

For the purpose of quality assessment and outcome reporting, we categorised *de novo* the 38 studies into three groups, according to the intended purpose or setting of the information:

• Category 1: Explaining medical or surgical procedures (17 studies);
• Category 2: Management of long-term conditions (11 studies);
• Category 3: Topics related to public health, health promotion or illness prevention (10 studies).

#### 3.2.1. Category 1: explaining medical or surgical procedures (17 studies)

**Figure 2:**
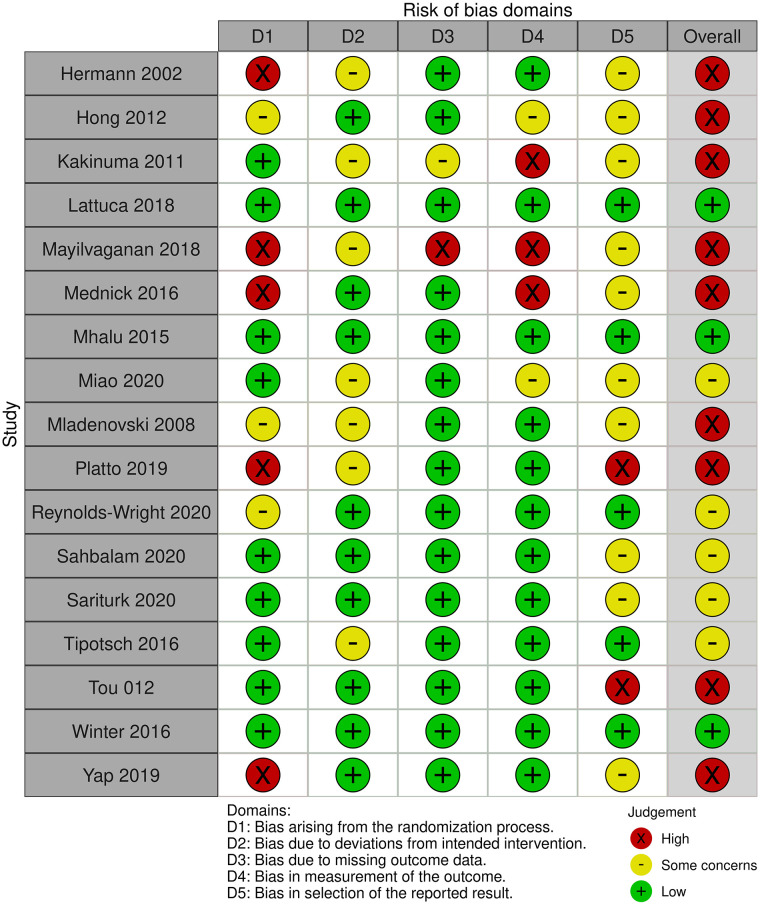
Risk of Bias in the Category 1 studies.

Figure 2 and Table 1 summarise the risk of bias judgements and findings across the studies in category 1 (17 studies, *n* = 2,655, sample range 30–843) (13, 18, 19, 26, 32–36, 38, 41–43, 47–49, 51).

Nine of the 17 studies were assessed as having a high risk of bias, most commonly due to the randomisation process. The other studies were rated as at low risk of bias (3 studies) (36, 43, 48) or having “some concerns” (5 studies), due to small sample sizes or a lack of protocol registration (33, 38, 41, 42, 49).

##### 3.2.1.1. Effects on knowledge

Knowledge was assessed in twelve studies and provision of an animation resulted in positive outcomes in eight of them (13, 19, 33, 35, 43, 48, 49, 51). From the eight studies in which outcomes favoured animation, four were related to informed consent (19, 33, 43, 49). In the remaining studies one study showed some benefits from the animation (i.e., favoured animation at one recruitment site and showed no difference between arms at the other two sites) (38)) and three studies showed no differences between the intervention and control arms (18, 32, 47). It is notable that knowledge outcomes favoured the animation in almost all studies (7/8) when the comparator was standard care or spoken information, but only in a minority of studies (1/4) when the comparator was a work of written information or static images.

No Category 1 study reported better knowledge outcomes in the control group (See Supplementary S2: Table 4 for a detailed summary).

##### 3.2.1.2. Effects on attitudes and cognition

Attitudes and cognitions were assessed in thirteen studies (18, 19, 26, 32–34, 38, 42, 43, 47–49, 51) and only two studies reported statistically significant differences in favour of the animation (19, 48).

Six studies reported no statistically significant differences between arms (32–34, 43, 49, 51), of which three were related to informed consent (33, 43, 49).

Four studies showed some benefits with animation (i.e., outcomes favoured animation in some items or sub-scores, but found no differences between arms with the remainder) (18, 26, 42, 47).

One study (38) showed mixed results: one recruitment site (out of three sites) reported in favour of standard care on the information being “very helpful” and the other two recruitment sites reported no difference between arms. All three sites reported no difference on information they received being “very clear”. Only one out of three recruitment sites reported in favour of animation on “information utility” (38).

##### 3.2.1.3. Effects on behaviours

Behaviours and skills were assessed in two studies (36, 41) and both studies reported in favour of the animation. One study reported that patients who watched the animation produced better quality sputum samples (36) and the other study the animation was more effective for preparing children for dental treatment (41).

#### 3.2.2. Category 2: management of long-term conditions (11 studies)

**Figure 3:**
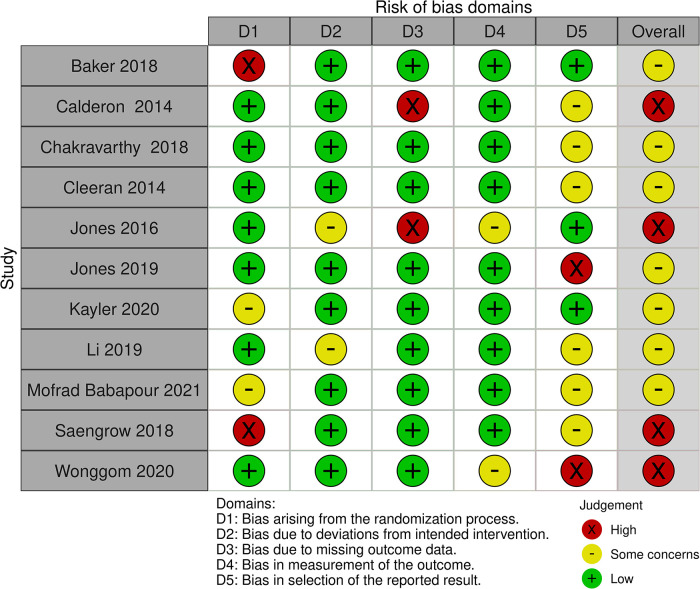
Risk of bias in the Category 2 studies.

Figure 3 and Table 2 summarise the risk of bias judgements and findings across studies in Category 2 (11 studies, *n* = 1238, range 36–240) (20, 21, 23–25, 27, 40, 44–46, 54).

Just over half of the studies (7/11) in this category were rated as having “some concerns” of bias due to unclear randomisation, small sample size or lack of protocol registration (20, 21, 24, 27, 44–46).

Four studies were rated as having a high risk of bias due to the randomisation process, missing data or being underpowered (through being unable to recruit the target sample size) (23, 25, 40, 54).

None of the studies in this category were rated as low risk of bias overall.

##### 3.2.2.1. Effects on knowledge

Knowledge was assessed in ten studies and provision of an animation resulted in positive outcomes in six of them (20, 21, 24, 40, 44, 45) and no difference between arms in one study (27).

The other three studies reported some benefits from the animation (i.e., favoured animation at 90 days but not at 30 days follow-up (54); favoured animations on only one of eight measures (25); and favoured animation in participants with inadequate functional health literacy but not in participants with marginal or adequate functional health literacy (23)).

No Category 2 study reported better knowledge outcomes in the control group (See Supplementary S3: Table 5 for a detailed summary).

##### 3.2.2.2. Effects on attitudes and cognitions

Attitudes and cognitions were assessed in five studies (20, 25, 44, 46, 52) and three of the studies reported no significant differences between arms (20, 44, 52). One study reported some improvement with animation on aspects of outcome measures: four out of 18 illness perception items; two out of four medication beliefs items, and cardiac anxiety items (25). The other study reported a positive effect of animation on the quality of recovery but no differences on: perceptions of surgery, recovery, mobilization and oral nutrition, or on traditional beliefs about recovery after surgery (46).

No Category 2 study reported better attitudes and cognitions outcomes in the control group.

##### 3.2.2.3. Effects on behaviours

Behaviours and skills were assessed in five studies (20, 21, 25, 40, 52) and only one of the five studies (into children with epilepsy) reported in favour of animation in terms of improved drug adherence (40). Two studies reported some benefits with animation (i.e., favoured animation on 1 out of 4 measures (25); favoured animation for IRD willingness only (20)). In the other two studies there was no reported difference between the intervention and control arms in terms of compliance and self-care behaviour (21, 52).

No Category 2 study reported better behaviour outcomes in the control group.

#### 3.2.3. Category 3: topics related to public health, health promotion or illness prevention (10 studies)

**Figure 4:**
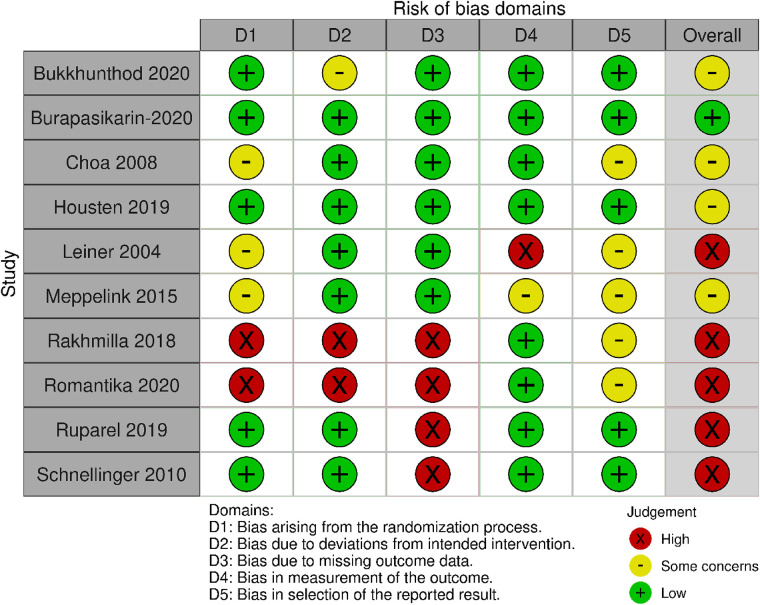
Risk of bias in the Category 3 studies.

Figure 4 and Table 3 summarise the risk of bias judgements and findings across the studies (10 studies, *n* = 1,753, sample range 80–270).

Five out of ten studies were rated as having a high risk of bias (8, 9, 37, 39, 50). The most common reasons were the randomisation process and missing outcome data. Four studies were rated as having some concerns, due to lack of protocol or sample size calculation (28–31). Only one study was rated as having low risk of bias (22).

##### 3.2.3.1. Effects on knowledge

Knowledge was assessed in eight studies (8, 9, 28, 30, 31, 37, 39, 50) and provision of an animation resulted in positive outcomes in five of them (9, 28, 37, 39, 50). The participants in those four out of five studies were either school children or mothers of young children (9, 28, 37, 50). Two studies showed some benefits from animations (i.e., favoured animation at 4 weeks follow-up but not immediately after the intervention (8); favoured animation in the low health literacy spoken text + animation group but not in the high health literacy group or the low health literacy written information + animation group (31)). One study reported no differences between arms regardless of participants’ level of health literacy (30).

No Category 3 study reported better knowledge outcomes in the control group (See Supplementary S4: Table 6 for a detailed summary).

##### 3.2.3.2. Effects on attitudes and cognitions

Attitudes and cognitions were assessed in three studies (8, 39, 50) and two studies reported significant differences in favour of animation (39, 50). In one study mothers of young children did not think they had learnt something new about the use of antibiotics by watching the animation when compared to those provided with a pamphlet. They also did not think the animation was more interesting or useful (8).

##### 3.2.3.3. Effects on behaviours

Behaviours and skills were assessed in two studies (22, 29). One study assessing use of long-acting reversible contraception (LARC) in postpartum women, reported in favour of video animation (22). The other study comparing live CPR instructions from a dispatcher over the phone and video animation reported better scores in checklist assessment and time interval compliance with the animation. However, differences were not apparent for the psychomotor skill measures (29).

## 4. Discussion

### 4.1. Summary of findings

This systematic review of trials of video animations as information tools for patients and the general public included 38 studies. Data pooling was not possible due to significant variation across aspects of the trials. Most trials assessed the effect of cartoons or 3D animations. Knowledge was the outcome most often assessed, usually very soon after participants had accessed information. There were consistently positive effects of animations on knowledge, particularly when compared to standard care or spoken information, but also when compared to easy-to-read information, standard printed information, real-time or static images, and audio-recorded information. Participants’ attitudes and cognitions were evaluated less frequently, showing benefits of animations in some studies but no clear benefits in as many studies. Patient behaviours were assessed least frequently, reporting animation benefits in half of relevant studies and no differences in the remainder. Across the 38 studies, only one reported statistically significant benefits of the control intervention over animation (38).

### 4.2. Strengths and limitations of the research

A number of processes were used in the systematic review to reduce potential for bias, including: protocol registration; multiple database searches; entry criteria; inclusion of non-English articles; citation searching; and dual decision-making on study inclusion, data extraction and risk of bias assessment. One strength of the findings was the breadth of health settings and country of origin: although most of the trials were undertaken in high income countries, there was a significant geographical spread.

The included studies were all real world, pragmatic evaluations of outcome effectiveness. However, they did not collect process data (such as attention monitoring or eye tracking), which could indicate patient engagement with the animations and provide insights into patterns of effectiveness. Furthermore, few trials assessed knowledge in the longer-term. In some settings, for example, management of long-term conditions or preventive health behaviours, longer-term knowledge increases would be a more important indicator of intervention success. However, in other settings, such as the preparation of patients for surgery or CT scanning, shorter-term knowledge gains would be valid indicators of effect.

Individual trials were often small and with substantial variation across a number of different study elements. Furthermore, the quality of the 38 trials was mixed, with only four trials rated as having low risk of bias. Frequent sources of risk of bias were randomisation processes, small sample size or lack of sample size calculation, missing outcome data, and lack of protocol publication. Half of the trials recruited fewer than 100 participants and most of these had no stated sample size calculation, which raises two legitimate concerns: (i) possible Type 2 statistical error in studies reporting null effects, and (ii) possible publication bias associated with studies reporting beneficial effects of animations.

Only a minority of articles provided a link to the tested animation. No doubt copyright restrictions were influential in several trials but the inability to play the evaluated animations does restrict the conclusions that can be drawn. For example, it makes it impossible to assess the content, tone, accessibility or quality of animations. Furthermore, it prevents study replication or the ability of build on effective interventions, both of which are crucial elements of scientific methods.

### 4.3. Implications of the findings

Overall, the findings were similar to those seen in uncontrolled studies (10–14). While the findings of this review suggest there is a potential role for animations as information tools, there remains a lack of good quality evidence on their effectiveness, as well as a lack of clarity on which types of animations and which animation elements are associated with optimal use, acceptability and effectiveness. This implies the need for three types of research:

• First, larger trials that are less susceptible to bias. It should be possible for trials to use allocation concealment when recruiting participants, even if blinding of outcome assessment is not possible. Trials using cluster allocation may be the solution to the inherent problems with intervention blinding in information research, although cluster trial design decisions are not straightforward. Sample size calculation is also essential, although it may be a lesser priority in feasibility or pilot trials. Also important are an adjustment for statistical multiplicity when multiple outcome measures are being assessed, and health economic analyses, particularly when animations are being provided instead of a lower cost information intervention.
• Second, implementation research, evaluating the use of animations in practice to assess the impact of context (particularly health setting and delivery) on uptake and effectiveness. For example, one advantage of animations over static images (in print or online) is that they can be dynamic, having potential to illustrate procedures, interventions and pathology in ways that other formats may not be able to do, which may make them particularly well suited to explaining complex procedures or treatment processes.
• Third, fine-grained process studies may be needed to assess the effects of animation length as well as various design elements on users' attention and knowledge acquisition. One concern is that animations may lead to, or even encourage surface level learning, rather than more meaningful or conceptual learning. Furthermore, users' attention to video and video animations may be limited; this has implications for more complex or detailed topics, when the useful function of animations could be limited to an introduction or overview.

It is vital that reports of future animation studies allow access to the evaluated animations, or it is impossible to discern quality or the effects of mediators (and so understand patterns of effectiveness and ineffectiveness) (16). Furthermore, reports should make clear the extent of patients' access to animations. In some healthcare settings, such as preparation for CT scanning, access will necessarily be time-limited. However, in many settings animations may be available online and with unlimited patient access. This situation can create a mismatch with access limits imposed within a controlled study environment; at the very least, this issue needs acknowledgment in study reports.

The included trials were a mix of studies of animations used in addition to other provision and those in which animations were a replacement. This is an important distinction and one that needs clarification in future studies, given the possible implications of healthcare services having to develop and deliver information in more than one format. Finally, animations may be most beneficial (in relative terms) for children and population groups with lower levels of education or health literacy, but currently the evidence base does not permit such an indication of relative effectiveness.

## 5. Conclusions

This is the first systematic review of the effectiveness of video animations as patient information tools, when evaluated in controlled studies. Our findings indicate mostly positive effects on knowledge, particularly in the short-term, and some positive effects on attitudes and cognitions. They also indicate mostly positive effects on behaviour, although this outcome was evaluated in only nine trials. There is almost no evidence of worse patient outcomes from animations.

## Data Availability

The original contributions presented in the study are included in the article/Supplementary Material, further inquiries can be directed to the corresponding author/s.
