# Supplementary material for: The effectiveness of video animations as information tools for patients and the general public: A systematic review
Source: Front Digit Health. 2022 Oct 31;4:1010779. doi: 10.3389/fdgth.2022.1010779 (PMC9910310; doi:10.3389/fdgth.2022.1010779)
Supplement: Supplementary file 1 [file Table1.docx]

# S1: Search Strategy

## Medline (via Ovid)

| **Database Version & Platform:** | Ovid MEDLINE(R) ALL <1946 to June 08, 2021> |
| --- | --- |
| **Date Range Searched:** | No limits by date. |
| **Date of Most Recent Search:** | 09/06/2021 |
| **Records Retrieved:** | 968 |

| **Line Number:** | **Search Strategy:** | **Hits:** |
| --- | --- | --- |
| 1 | Cartoons as Topic/ | 624 |
| 2 | Caricatures as Topic/ | 282 |
| 3 | Motion Pictures/ | 8059 |
| 4 | ((animation* or computer-animat* or digital-animat* or digitally-animat* or motion comic* or motion-comic* or anime) not "suspended animation").ti,ab,kw,kf. | 3164 |
| 5 | (animated adj6 (film* or video* or visual* or picture* or image* or character*1 or avatar* or cartoon* or webtoon* or web-toon* or web-cartoon* or comic* or web-comic* or webcomic* or caricature* or manga)).ti,ab,kw,kf. | 729 |
| 6 | ((computer* or digital*) adj4 (animat* or character*1 or avatar* or cartoon* or webtoon* or web-toon* or web-cartoon* or caricature*)).ti,ab,kw,kf. | 783 |
| 7 | (illustrated adj3 (film* or video* or watch*)).ti,ab,kw,kf. | 174 |
| 8 | ((avatar* or cartoon* or webtoon* or web-toon* or web-cartoon* or caricature*) and (film* or watch* or view* or video*)).ti,ab,kw,kf. | 839 |
| 9 | or/1-8 | 13561 |
| 10 | exp Health/ | 387525 |
| 11 | exp Public Health/ | 8254670 |
| 12 | exp Population Health/ | 39607 |
| 13 | exp Health Education/ | 250960 |
| 14 | exp "Patient Education as Topic"/ | 87155 |
| 15 | exp Consumer Health Information/ | 10693 |
| 16 | Health Communication/ | 2730 |
| 17 | Attitude to Health/ | 84769 |
| 18 | Patient Acceptance of Health Care/ | 50194 |
| 19 | exp Education/ | 836317 |
| 20 | exp Teaching/ | 88352 |
| 21 | exp Curriculum/ | 88715 |
| 22 | exp Teaching Materials/ | 120829 |
| 23 | exp Education, Medical/ | 171376 |
| 24 | exp Education, Public Health Professional/ | 805 |
| 25 | exp Education, Graduate/ | 89198 |
| 26 | exp Education, Dental/ | 19706 |
| 27 | exp Education, Nursing/ | 84970 |
| 28 | exp Education, Pharmacy/ | 8288 |
| 29 | (health* adj3 (educat* or public* or population* or communit* or knowledge* or literate or literacy or attitude*)).ti,ab,kw,kf. | 499417 |
| 30 | (curricul* or school* or pedagog* or andragog* or tuition or tutelage or tutorial* or tutor* or class* or lesson* or lecture* or seminar*).ti,ab,kw,kf. | 1886339 |
| 31 | (inform*3 or instruct* or self-instruct* or learn* or e-learn* or self-learn* or educat* or self-educat* or teach* or train* or taught or self-taught or counsel* or advice or advis* or guide* or guidance or understand* or knowledge* or self-knowledge).ti,ab,kw,kf. | 4186495 |
| 32 | or/10-31 | 12251228 |
| 33 | 9 and 32 | 11433 |
| 34 | Randomized Controlled Trials as Topic/ | 144590 |
| 35 | Randomized Controlled Trial/ | 533119 |
| 36 | Random Allocation/ | 105441 |
| 37 | Double-Blind Method/ | 164877 |
| 38 | Single-Blind Method/ | 30319 |
| 39 | Clinical Trial/ | 529217 |
| 40 | clinical trial, phase i.pt. | 21725 |
| 41 | clinical trial, phase ii.pt. | 34992 |
| 42 | clinical trial, phase iii.pt. | 18505 |
| 43 | clinical trial, phase iv.pt. | 2111 |
| 44 | controlled clinical trial.pt. | 94203 |
| 45 | randomized controlled trial.pt. | 533119 |
| 46 | multicenter study.pt. | 295976 |
| 47 | clinical trial.pt. | 529217 |
| 48 | exp "Clinical Trials as Topic"/ | 358441 |
| 49 | or/34-48 | 1433932 |
| 50 | (clinical adj trial$).tw. | 400977 |
| 51 | ((singl$ or doubl$ or treb$ or tripl$) adj (blind$3 or mask$3)).tw. | 180636 |
| 52 | Placebos/ | 35522 |
| 53 | placebo$.tw. | 225747 |
| 54 | randomly allocated.tw. | 31163 |
| 55 | (allocated adj2 random$).tw. | 34599 |
| 56 | or/50-55 | 681528 |
| 57 | Research Design/ | 113177 |
| 58 | (experiment* adj5 (research* or design* or group* or psychology)).tw,kw. | 194541 |
| 59 | (experiment*1 adj3 control*1 adj8 (group* or divide* or assign* or allocate* or random*)).tw,kw. | 1597 |
| 60 | (control* adj3 condition*).tw,kw. | 54619 |
| 61 | (research adj4 design*).tw,kw. | 48885 |
| 62 | (DoE adj6 (experiment* or approach*)).tw,kw. | 1188 |
| 63 | (counterbalanc* or counter-balanc* or "counter balanc*").tw,kw. | 13243 |
| 64 | (latin square* or latin-square*).tw,kw. | 5345 |
| 65 | ((independent or repeated) adj3 measure*).tw,kw. | 65087 |
| 66 | (independent-measure* or repeated-measure*).tw,kw. | 52275 |
| 67 | (between-subject* or "between subject*" or within-subject* or "within subject*" or between-group* or "between group*").tw,kw. | 169282 |
| 68 | (matched pair* or matched-pair*).tw,kw. | 10257 |
| 69 | (mixed adj4 (research* or method*)).tw,kw. | 32664 |
| 70 | (multiple adj4 method*).tw,kw. | 23990 |
| 71 | (mixed-method* or multiple-method* or multi-method* or multimethod*).tw,kw. | 34109 |
| 72 | ((closed ended or closed-ended) and (open ended or open-ended)).tw,kw. | 497 |
| 73 | (qualitative* and quantitative*).tw,kw. | 102886 |
| 74 | or/57-73 | 786146 |
| 75 | 33 and (49 or 56 or 74) | 991 |
| 76 | letter/ | 1138764 |
| 77 | editorial/ | 570065 |
| 78 | news/ | 207370 |
| 79 | exp historical article/ | 403073 |
| 80 | comment/ | 913133 |
| 81 | (letter or comment*).ti. | 165754 |
| 82 | (comment or conference or letter).pt. | 1576641 |
| 83 | or/76-82 | 2603353 |
| 84 | 75 not 83 | **968** |
