# Supplementary material for: The effectiveness of video animations as information tools for patients and the general public: A systematic review
Source: Front Digit Health. 2022 Oct 31;4:1010779. doi: 10.3389/fdgth.2022.1010779 (PMC9910310; doi:10.3389/fdgth.2022.1010779)
Supplement: Supplementary file 2 [file Table2.docx]

**The effectiveness of video animations as informational tools for patients: A systematic review**

# Supplementary material

### Table 2: category 1 - Explaining medical or surgical procedures

| **Authors, years, country** | **Study design** | **Participants, Setting, Education level between the 2 groups** | **Age Mean (SD)/ % Male** | **Total sample;**  **Intervention descriptor & sample size (I);**  **Control descriptor & sample size (C)** | **Intervention details and link(if provided)** | **Results (Intervention vs Control)** (**Knowledge = knowledge or understanding. Attitudes & Cognitions = satisfaction or self-efficacy or confidence in decision, etc. Behaviours = behaviours or skills or intended behaviours) , p value**  | | | **Group favoured** |
| --- | --- | --- | --- | --- | --- | --- | --- | --- | --- |
|  |  |  |  |  |  | **Knowledge** | **Attitudes & Cognitions** | **Behaviours** |  |
| Hermann, 2002, Austria{Hermann, 2002 #2004} | RCT | Patients undergoing thyroid surgery  Education level NR | Age: 56.2 (NR)  Male: 22% | N= 80  I=36  3D computer animation  C=44  Written text | 7 minutes 3D animation which portrayed the process of thyroid surgery  Watched once on the VHS video player  Link NR | Knowledge (of risks), mean score (SD):  I: 2.3 (1.2)  C: 2.2 (1.3)  P= NS  Knowledge (of complications), mean score (SD):  I: 2.9 (SD NR)  C: 2.8 (SD NR)  P= NS | Desire to know how the surgical procedure is carried out, means score out of 5 (SD):  I: 4.3 (1.3)  C: 3.7 (1.4)  P= NS  Have better understanding of the surgical steps, mean score out of 5 (SD):  I: 4.5 (1.0)  C: 3.9 (1.2)  P= 0.018  Portrayal was lifelike, means score out of 5 (SD):  I: 4.6 (1.2)  C: 3.8 (1.2)  P=0.0008  Fear of surgery was reduced, means score (SD):  I: 4.1 (1.1)  C: 3.0 (1.3)  P=0.00019  Comfort with procedure (“the inner yes”), mean score (SD):  I: 4.5 (0.7)  C: 3.8 (SD NR)  P=0.004  Professional competence, mean score (SD):  I: 4.9 (0.2)  C: 4.7 (0.7)  P=NS  Would like to see real video of surgery, means score (SD):  I: 2.4 (1.8)  C: 2.8 (1.8)  P=NS |  | **Knowledge:** No difference between arms  **Attitudes & Cognitions:**  Favours animation (4 out 7 items; no difference between arms on 3 out 7 items) |
| Hong, 2012, Korea{Hong, 2012 #1762} | RCT | ED patients about to undergo CT scan  No statistical significant difference in education level | Age: I= 38 (13.7); C= 42.3 (14.0),  Male: I= 45%,C=56% | N= 150  I=75  Animation-assisted informed consent  C=75  Standard verbal informed consent | 7 minutes animation assisted informed consent about the contrast Computed Tomography (CT)scan  Watched once on tablet computer  Link NR | Knowledge, combined mean score (SD):  I: 8.6 (1.4)  C: 8.2 (1.5)  I vs C: p=NS  Individual knowledge items, mean score (SD):  Purpose:  I: 8.9 (1.5)  C: 8.3 (1.9)  I vs C: p=0.026  Process & method:  I: 8.9 (1.4)  C: 8.4 (1.8)  I vs C: p=0.048  Notice:  I: 8.8 (1.6)  C: 8.4 (1.8)  I vs C: p=NS  Complications:  I: 8.4 (1.8)  C: 8.2 (2.0)  I vs C: p=NS  Alternative:  I: 8.0 (2.2)  C: 7.4 (3.0)  I vs C: p=NS | Satisfaction, combined mean (SD):  I: 8.7 (1.3)  C: 8.1 (1.9)  P=0.0.45  Individual satisfaction items, mean (SD):  Speed of explanation:  I: 8.6 (1.7)  C: 8.0 (2.1)  P=0.048  Familiarity:  I: 8.8 (1.7)  C: 8.2 (2.1)  P= 0.048  Overall satisfaction:  I: 8.8 (1.4)  C: 8.1 (2.2)  P=0.038 |  | **Knowledge:** favours animation.  **Attitudes & Cognitions** (satisfaction): favours animation. |
| Kakinuma 2011, Japan{Kakinuma, 2011 #794} | RCT | Patients about to undergo surgery for cancer  No statistical significant difference in education level | Age: I= 60.1 (13.7), C= 60.0 (13.2),  Male: 59.2% | N=211  I= 106  Cartoon animation+ visit from anesthesiologist  C= visit from anesthesiologist | 30 minutes (several brief sections which lasted 1 to 2 minutes) cartoon animation of the role of anaesthesiologists during surgery, gave nil per os (NPO) instructions, and the rationale for the NPO instructions, usual general or combined general and epidural anaesthesia procedures, and postoperative recovery  Patients were allowed to repeat any part of the video as many times as they wanted within 30 minutes.  Watched on laptop, frequency NR  Link NR | **Knowledge mean(SD**):  I: 12.5 (1.4)  C: 11.2 (1.7)  I vs C: p< 0.0001 |  |  | **Knowledge**:  Favours animation |
| Lattuca 2018, France{Lattuca, 2018 #6333} | RCT | Patients undergoing coronary angiography and/or angioplasty,  Cardiology unit (39 participating centres in France)  No statistical significant difference in education level | Age: 67.3 (11.6),  Male: 72% | N= 843  I=424  3D educational video + standard information  C=419  Standard information alone | 5 minutes 3D education video on the procedure of the coronary angiography  Watched once on a tablet  <https://ars.els-cdn.com/content/image/1-s2.0-S0002870318300784-mmc1.mp4> | Knowledge, mean total score out of 16 (SD):  I: 11.8 (2.8)  C: 9.5 (3.1)  P<0.001  Knowledge, higher scores in I arm for all 6 sub-scale scores (p<0.001) | Satisfaction with information, mean score out of 10 (SD):  I: 8.4 (1.9)  C: 7.7 (2.3)  P<0.001 | Not assessed | **Knowledge:** favours animation.  **Attitudes & Cognitions:** favours animation. |
| Mayilvaganan 2018, India{Mayilvaganan, 2018 #1393} | RCT (3 Arms) | patients who underwent hemithyroidectomy  Education level NR | Age: Group 1 static images 31.2 (13.3), Group 2 3D model 35.4 (12.8), Group 3 Video 36.6 (11.8),  Male: 25% | N=60  I1= 20  Animated video  I2= 20  3D thyroid model  C= 20  Conventional diagram | 2 minutes animated cartoon video of the hemithyroidectomy procedure  Animation frequency and delivery NR  IJEM-22-520-v001.mp4 |  | **Patient Satisfaction Questionnaire:**  Experience with the model, mean (SD):  Group 1: 1.8 (0.4);  Group 2: 1.7 (0.6);  Group 3: 2.5 (0.4).  P<.01  Overall satisfaction, mean (SD):  Group 1: 2.1 (1.1);  Group 2: 1.9 (1.0);  Group 3: 2.0 (0.7).  P=NS  **Unmet informational needs were met:**  Group 1: 50% patients;  Group 2: 65% patients;  Group 3: 75% patients.    Group 3 vs Group 1: p=0.12  Group 3 vs Group 2: p=NS |  | **Attitudes & Cognitions:** favours animation on one item.  No differences between groups on other items or overall score. |
| Mednick 2016, Canada{Mednick, 2016 #517} | RCT | Patients undergoing an initial IVFA investigation  Education level NR | Age: 66.9 (12.4),  Male:56% | N= 52  I= 26  Narrated white board animation    C1= 26  Standard consent  C2= 26  Standard consent + narrated white board animation | 3.5 minutes narrated white board animation of the procedure of intravenous fluorescein angiography(IVFA)  Watched on computer, frequency NR  Link NR | **Knowledge, mean score out of 6 (SD):**  I: 5.0 (0.9)  C1: 4.4 (1.1)  C2: 5.6 (0.6)  I vs C1: p=0.023;  C2 vs C1: p<.001  C2 vs I: p=NS | Satisfaction:  3 questions:  (1) fully understood what IVFA entailed; (2) felt well informed on consent; (3) understood the thoroughness of consent process:  I vs C1; all 3 NS |  | **Knowledge:**  Favours animation, and combined animation + standard consent.  **Attitudes and Cognitions:**  No differences between arms |
| Mhalu 2015, Tanzania{Mhalu, 2015 #6334} | RCT | Presumptive TB patients, Outpatient department in Tanzania  Education level NR | Age median(range): 39.1 (37.0 to 50.0);  Male: 53% | N= 200  I= 100  video animation    C= 100  Standard care | 4 minutes animated sputum submission instructional video  Watched once on a laptop  <https://www.youtube.com/watch?v=2sd2d2_pNBA> | Not assessed | Not assessed | Quality of sputum sample:  I: %NR  C: %NR  P<0.0001  Sputum volume adequate:  I: 78%  C: 45%  P<0.0001  Salivary sample provided (i.e. incorrectly):  I: 14%  C: 39%  P=0.0001 | **Behaviours:** favours animation |
| Miao 2020, Australia{Miao, 2020 #6335} | RCT | patients referred for Mohs micrographic surgery, 2 large outpatient dermatology facilities in Sydney, Australia  No statistical significant difference in education level | Age: I= 63.1 (10.9), C= 65.1 (13.0),  Male 55% | N=102  I= 51  Video-assisted animation consent  C=51  Standard consent | 5.50 minutes video animation which outlined the standard consent for Mohs micrographic surgery. The video was designed to supplement rather than replace the patient’s consultation with his or her physician.  Watched on iPad, Frequency NR  Link NR | Knowledge, means score out of 10 (SD):  I: 8.6 (1.8)  C: 6.3 (2.6)  I vs C: p=0.02  The intervention arm had higher knowledge scores on 8/10 individual items (at p=NS). No difference between arms on 2/10 items (p=NS) | Satisfaction, mean score out of 35 (SD):  I: 31.5 (3.1)  C: 30.1 (4.4)  I vs C: p= NS  The intervention arm had higher satisfaction scores on 1/7 items (p=NS) with no difference between arms on 6/7 items (p=NS) | Not assessed | **Knowledge:** favours animation  **Attitudes & Cognitions:**  No difference between arms. |
| Mladenovski 2008, New Zealand{Mladenovski, 2008 #871} | RCT | Patients referred to the University of Otago School of Dentistry for third molar extraction  Education level NR | Age (range): 16 to 49yrs  Male: 25% | N= 30  I=16  Multimedia  C=14  Leaflet | 6 minutes animation of the third molar extraction 3D programme. The multimedia software employed audio and visual files on a CD-ROM  Watched on computer  Link NR | Knowledge, 14 items:  No differences between arms on any of the 14 items (all p=NS) | Self-rated knowledge:  % rated themselves as ‘excellent or very good or good’:  I: 62.5%  C: 38.5%  I vs C: p<.05  Satisfaction ratings of intervention, 12 items, (% agreeing):  ‘Helpful’  I: 93.8%  C: 15.4%  P<.05  ‘Preferred’  I: 75.0%  C: 15.4%  P<.05  No difference between arms on other 10/12 items. | Not assessed | **Knowledge:** no difference between arms  **Attitudes & Cognitions:**  Self-rated knowledge favours animation;  Satisfaction favours animation on 2/12 items; no difference between arms on 10/12 items. |
| Platto 2019, USA{Platto, 2019 #347} | RCT | Patients awaiting dermatologic surgery  Education level NR | NR | N= 45  I=22  Video animation + conventional physician consultation  C= 23  Convention physician consultation alone | 2 minute animated educational video covering anaesthesia, excision, repair , post-operative wound care, and pain management  Frequency and delivery NR  <https://www.youtube.com/watch?v=x7ujWviMNxM> |  | Want additional information about procedure, mean (SD):  I: 5.1 (2.0)  C: 6.3 (3.1)  P=NS |  | **Attitudes & Cognitions:** desire for more information: no difference between arms |
| Reynolds-Wright 2020, UK {Reynolds-Wright, 2020 #294} | RCT and Quasi RCT Paris site | Gynaecological patients with confirmed gestation in abortion clinics in three locations: Paris, Stockholm and Edinburgh  Education level NR | Age: I= 28 (NR), C= 27.8 (NR)  Male: 0% | N= 172  I=104  Animation  C= 68  Standard care | 3 minute video animation summarised the process of early medical abortion (EMA) using simple language and animated characters representing women of diverse ages and ethnicities  Watched once on a laptop computer in a private room in the clinic  <https://vimeo.com/302663935> | Knowledge, mean score out of 8 (SD):  Site 1:  I: 3.5  C: 3.3  P=0.624  Site 2:  I: 3.9  C: 2.9  P=0.007  Site 3:  I: 5.3  C: 5.1  P=0.61 | Information ‘very helpful’: Site 1:  I: 97%  C: 93%  P=NS  Site 2:  I: 74%  C: 100%  P=0.001  Site 3:  I: 79%  C: 64%  P=NS  Information ‘very clear’:  Site 1:  I: 91%  C: 93%  P=NS  Site 2:  I: 89%  C: 77%  PNS  Site 3:  I: 79%  C: 64%  P=NS  Information utility (rated 10/10):  Site 1:  I: 76%  C: 60%  P=NS  Site 2:  I: 86%  C: 67%  P=0.048  Site 3:  I: 68%  C: 64%  P=NS | Not assessed | **Knowledge:** favours animation at 1/3 sites. No difference between arms at 2/3 sites.  **Attitudes & Cognitions:**  Favours **standard care** at 1/3 sites on information ‘very helpful’. No difference between arms at 2/3 sites.  No difference between arms on information ‘very clear’ at all 3 sites.  Favours animation on utility at 1/3 sites. No difference between arms at 2/3 sites. |
| Sahebalam, 2020, Iran {Sahebalam, 2020 #234} | RCT | Healthy Pediatric dental participants in the clinical department of pediatric dentistry in Iran  Similar level, all in Primary school | Age: 5.23 (0.63), Male: 35.4% | N= 50  I= 25  Video animation  C= 25  Tell-Show-Do technique | 1.14 minutes animation called: Jilo goes to a dentist. Dental instruments and procedures are designed in the form of objects and concepts that a child can understand, and that involve their imagination. For example, the suction procedure has been modelled using an elephant's trunk and its power to suck in water  Watched once  <https://www.aparat.com/v/g7wFt> | Not assessed | Not assessed | Non-cooperation at 1^st^ visit, mean score out of 5 (SD):  I: 0.3 (0.6)  C: 1.0 (1.1)  I vs C: p=0.015  Non-cooperation at 2^nd^ visit, mean score out of 5 (SD):  I: 0.6 (0.9)  C: 1.3 (1.1)  I vs C: p=0.019 | **Behaviour:** favours animation. |
| Sariturk, 2017, Turkey {Sariturk, 2017 #492} | RCT | Adult patients who were scheduled to undergo autologous or allogeneic peripheral stem cell transplantation (SCT) at the Adana Bone Marrow Transplantation Unit of Baskent University Faculty of Medicine and donors from whom peripheral stem cell collection for allogeneic transplantation was planned  No statistical significant difference in education level | Age: 47 (14),  Male: 56.1% | N= 82  I= 42  Audio-visual + standard verbal + written information  C=40  standard verbal + written information | 10 minutes information animation covered disease status, purpose of the treatment, treatment principles, stem cell collection procedure, pre-treatment assessment, the drugs used and their side effects, infusion of stem cells, benefits expected from the treatment, treatment risks and side effects, other treatment options, and disposal of the cellular product  Watched once  Link NR | Not assessed | Satisfaction, overall mean out of 200 (SD):  I: 185.7 (22.2)  C: 183.8 (17.2)  I vs C: p=NS  Satisfaction with written informed consent sub-score, mean score out of 70 (SD):  I: 64.2 (8.5)  C: 60.3 (8.2)  I vs C: p=0.039  Satisfaction with informing doctor sub-score out of 70 (SD):  I: 64.7 (8.6)  C: 67.0 (5.8)  I vs C: p=NS | Not assessed | **Attitudes & Cognitions:** one sub-score favours animation; one sub-score no difference between arms. |
| Tipotsch-2016, Austria {Tipotsch-Maca, 2016 #538} | RCT | Patients who were scheduled for surgery for age-related cataract in Hietzing Hospital, Vienna  Education level differences between groups were NR | Age: 71 (7),  Male: 41% | N= 123  I=59  Computer animated video + face-to-face consultation + brochure  C= 64  Face-to-face consultation and a brochure | 6 minutes computer-animated video (Eyemaginations 3D-Eye, Eyemaginations) of the cataract surgery which covers covers background information, surgery, complications, post-op therapy and controls  Frequency and delivery NR.  Link NR | Knowledge, mean score out of 10 (SD):  I: 8.2 (0.5)  C: 7.2 (0.7)  I vs C: p=0.002  Animation arm had higher score on 4/10 items (p<.05) with no difference between arms on other 6/10 items. | Satisfaction with consent, mean score out of 5 (SD):  I: 1.1 (0.4)  C: 1.0 (0.2)  I vs C: p=NS  (low score = more satisfied) | Not assessed | **Knowledge:** favours animation.  **Attitudes & Cognitions:** no difference between arms. |
| Tou, 2013, Australia {Tou, 2013 #700} | RCT | Colorectal Unit at the Queen Elizabeth Hospital, Adelaide, Australia  Education level NR | Age: 59 (31),  Male: 39% | N= 31  I=16  Cartoon animated video + information sheet  C=15  Information sheet alone | 13 minutes 2D cartoon animation about bowel surgery including pre-, peri- and postoperative care  Watched the animation film on a desktop computer in a quiet room, frequency NR  Link NR | Knowledge, mean score (SD):  I: 5.8 (0.5)  C: 5.8 (0.6)  I vs C: p=NS | Satisfaction, mean score (SD):  On day of surgery:  I: 58.1 (27.7)  C: 49.5 (30.3)  I vs C: p=NS  Satisfaction, mean score (SD): 1 day post-op:  I: 38.1 (21.2)  C: 29.7 (23.8)  I vs C: p=NS  Satisfaction, mean score (SD): pre-discharge:  I: 25.6 (20.0)  C: 30.8 (25.3)  I vs C: p=NS | Not assessed | **Knowledge**: no difference between arms**.**  **Attitudes & Cognitions:** no difference between arms. |
| Winter 2016, Australia{Winter, 2016 #1528} | RCT | Patients presenting with acute renal renal colic to a public hospital (two teaching hosptials in Australia) who required a ureteric stent.  No statistical significant difference in education level | Age: 54 (NR)  Male: 75% | N= 92  I=47  Audio-visual presentation with cartoon animation  C=45  standard verbal consent | 7:07 minutes cartoon visual animation regarding the consent process for cystoscopy and insertion of ureteric stent  Watched on iPad, Frequency NR  <https://bjui-journals.onlinelibrary.wiley.com/doi/10.1111/bju.13595> | Knowledge, mean score (95% CI) out of 28:  I: 23.3 (22.2-24.3)  C: 20.1 (18.6-21.6)  I vs C: p<0.001 | Satisfaction, mean score (95% CI) out of 32:  I: 30.2 (29.4-31.0)  C: 29.1 (28.0-30.2)  I vs C: p=NS |  | **Knowledge:** favours animation.  **Attitudes & Cognitions:** satisfaction no difference between arms. |
| Yap, 2020, Singapore {Yap, 2020 #6336}} | RCT (3:1) | Consecutive patients undergoing coronary angiography and/or angio plasty were recruited from tertiary cardiac institution  The intervention group had significantly lower education levels compared with the control group (p = .024) | Age: 59.0 (94);  Male: 86% | N= 332  I= 252  Video + routine care  C=80  Routine care | 3 minutes animated patient education video of the coronary angiography and/or angioplasty procedure  Watched on smart phones, tablets as well as computers  Frequency NR  <https://youtu.be/R8AdaIbNq7Y> | Knowledge, mean score out of 12 (SD):  I: 10.2 (1.7)  C: 8.5 (2.9)  P<0.001 | Not assessed | Not assessed | **Knowledge:** favours animation. |

### Table 2: Category 2 - Management of long-term conditions

| **Authors, years, country** | **Study design** | **Participants, Setting, Education level between the 2 groups** | **Age Mean (SD)/ % Male** | **Total sample;**  **Intervention descriptor & sample size (I);**  **Control descriptor & sample size (C)** | **Intervention details and link(if provided)** | **Results (Intervention vs Control)** (**Knowledge = knowledge or understanding. Attitudes & Cognitions = satisfaction or self-efficacy or confidence in decision, etc. Behaviours = behaviours or skills or intended behaviours) , p value**  | | | **Group favoured** | |
| --- | --- | --- | --- | --- | --- | --- | --- | --- | --- | --- |
|  |  |  |  |  |  | **Knowledge** | **Attitudes & Cognitions** | **Behaviours** |  |  |
| Baker 2018, USA {Baker, 2018 #6330} | RCT | Patients undergoing anorectal testing for chronic constipation  No statistical significant difference in education level | Age: 47.3 (16).  Male: 16% | N= 100  I=50  Animated educational video  C=50  Traditional written educational pamphlet | 2 minutes animated videos (3 short videos about improving chronic constipation  Watched once on PC  <https://mygi.health/education/symptoms/constipation> | **Knowledge, mean score out of 13 (SD):**  I: 11.2 (1.8)  C: 11.1 (1.8)  I vs C: p=NS |  |  | **Knowledge:** no difference between interventions. | |
| Calderon 2014, {Calderon, 2014 #631} USA | RCT | Latino/Hispanic patients who were diagnosed with T2D at South Central Family Health Center (SCFHC) and the Charles Drew University of Medicine and Science Center for Health Services Research (Drew)  No statistical significant difference in education level | Age (Range) : 18 to >60  Male 18.3% | N= 240  I=118 Animated video  C=122 Control (easy-to read text) | 13 minutes animated video which featured an animated icon named “Corazón Quelate” (heart that beats; Spanish version) / “Lotta Hart” (English version). Corazón/Lotta engages viewers with an invitation into her home and emphatically shares her experience with diabetes.  Frequency NR  Link NR | **Change in adjusted DHLS mean(%):** 113 (55%) vs 109 (53%), F = 4.7, df = 1, P = 0.03  **Change in DHLS score in participants with inadequate functional health literacy (STOFHLA scores < 17) mean (%):**  61 (53%) vs 52 (50%); F = 7.12, p = .009  **Change in DHLS score in participants with marginal or adequate functional health literacy (STOFHLA scores ≥ 17 mean(%):** 37 (58%) and 50 (57%), F = 0.82, p =NS |  |  | **Knowledge**:  Favours animation overall , in particular  in participants with inadequate functional health literacy.  No difference in participants with marginal or adequate functional health literacy | |
| Chakravarthy 2018, USA {Chakravarthy, 2018 #445} | RCT | Emergency department of a large, urban academic hospital in USA  Education level NR | Age: I=41 (NR), C=34 (NR)  Male: NR | N= 52  I= 25  Video discharge instruction on opioid safety and proper usage, storage, and disposal + Standard care  C= 27  Standard care | 6-minute animated video on proper usage of opioids in addition to standard of care  Frequency NR  https://youtu.be/5FYNBvgmdsE | **Knowledge acquisition** **of opioid education [correct answer out of 26 (%)]:**  21.2 (82%) vs 16.8 (65%), p=0.001, Cohen's d 0.92 |  |  | **Knowledge:** Favours animation | |
| Cleeren 2014, Belgium {Cleeren, 2014 #651} | RCT | Dental practice located in Kester, Belgium  No statistical significant difference in education level | Age: 54.4(10.3),  Male: 29%. | N= 67  I=33  3D animation giving a general view of periodontitis  C=34  Real-time sketches of a general view of periodontitis | 6.20 minutes 3 D animated video about the general view of periodontitis beginning with the periodontal anatomy followed by the causes, symptoms, development and treatment of the disease  Patients were allowed to pause and rewind the videos on the computer screen during the clinic visit  Link NR | **knowledge on periodontitis mean(SD):**  post-test:  I: 8.42 (1.1)  C: 7.1 (1.6), p<.001  2 weeks follow up:  I: 7.5 (1.0)  C: 4.9 (1.3) , p<0.001  **Knowledge recall (mean difference from baseline):**  post-test:  -0.4 vs -1.3 (p<0.001)  2 weeks follow up: 1.0 vs 2.2 (p=0.03) |  |  | **Knowledge:** Favours animation | |
| Jones 2016, New Zealand {Jones, 2016 #551} | RCT | Patients with acute coronary syndrome  Education level NR | Age: 60.9 (10.2),  Male:70% | N= 70  I= 35 Animated video + standard care  C= 35  Standard care | 15 minutes, 2 parts animated video watched on iPad: (1) pathogenesis of acute coronary syndrome and resultant effects; (2) informing patients about behaviours to maintain health  Patients were given a website link where they could view the intervention again if desired  Link NR | Knowledge:  Naming adherence as heart healthy behaviour (post-intervention):  I: 55% (18/33)  C: 29% (9/31)  P=0.039  No difference between groups on naming 3 other heart healthy behaviours.  Naming adherence as heart healthy behaviour (7 weeks follow-up):  I: 50%(15/30)  C: 27%(8/30)  P=NS  No difference between groups on naming 3 other heart healthy behaviours. | Illness Perceptions:  (post-intervention):  I: beliefs +0.4 (95% CI -0.2 to 1.0)  C: beliefs -0.6 (95% CI -1.2 to 0.0), p=0.025  No differences between groups on the other 8 items of the IPQ.  7 weeks follow-up:  Intervention group had greater improved changes in scores on 3/9 items (p<.05) compared to Control group.  No difference in change scores between Intervention and Control groups on other 6/9 items.  Medication beliefs:  (Post-intervention):  Intervention greater change in specific concerns. I: -1.45 (95% CI -2.5 to -0.4) C : -0.3 (-0.8 to 1.3), p=0.031.  Intervention greater change in general-harm beliefs I: -0.5 (-1.2 to 0.2)  C 0.6 (-0.1 to 1.3). p=0.032  7 weeks follow-up, no differences between Intervention and Control groups.  Cardiac Anxiety avoidance:  7 weeks follow-up:  Intervention group had lower scores:  I: 6.6 (5.0 to 8.1).  C: 8.9 (7.3 to 10.4). p=0.038 | Return to work: no difference in number of days before return to work, p=NS  Return to normal activities  (number of days):  I: 17.4 (10-9 to 23.8)  C: 26.4 (20.7 to 32.1), p=0.043.  Exercise taken (minutes until 7 weeks follow-up):  I: +46.6 (7.6 to 82.5)  C: -9.2 (-48.2 to 29.7), p=0.049.  Self-reported medication adherence (7 weeks follow-up):  I: 46.9 (45.3 to 48.5)  C: 47.1 (45.4 to 48.7),  p=NS. | **Knowledge:** favours animation on 1 measure. No difference between arms on other 7 measures.  **Attitudes & Cognitions:**  Favours animation on 4/18 Illness Perception items. No difference between arms on other Illness Perception items.  Favours animation on 2/4 medication beliefs. No difference between arms on other items.  Favours animation on cardiac anxiety avoidance.  **Behaviours:** Favours animation on 1 / 4 measures.  No difference between arms on 3 / 4 measures. | |
| Jones 2019, New Zealand {Jones, 2019 #321} | RCT (3 Arms) | Colorectal & gynaecology oncology surgery patients  Education level differences between groups NR | Age: 58.7 (16.5)  Male: 37% | N= 96  I1= 33  Animations+ standard care  I2= 32  Active control (same as intervention without animations)+ standard care    C2= 31  Standard postoperative care | 10 minutes animated video to improve Postoperative Mobilization which described the purpose of early mobilization, the importance of early oral nutrition, and the  link between these two behaviors (actors filmed plus anatomical 2D and 3D)  delivered on a computer tablet at their bedside, frequency NR  https://youtu.be/1jmgAb1OTew |  | Perceptions of surgery and recovery, perceptions of early mobilization and early oral nutrition and traditional surgery recovery beliefs: (all p=NS),  Quality of recovery: (I1 vs C) p=NS  Quality of recovery: (I2 vs C) p=NS |  | **Attitudes & cognitions:** No difference between the 3 groups in Perceptions of surgery and recovery, perceptions of early mobilization and early oral nutrition and traditional surgery recovery beliefs.  Favours animation in terms of the quality of recovery compared to control | |
| Kayler 2020, USA {Kayler, 2020 #237} | RCT | Patient and care givers, Erie County Medical Centre (New York)  No statistical significant difference in education | Age: 60 (NR),  Male:  68% (patients only) | N=80,  I=42  Animation + audio recorded standard transplant nurse education  C= 38  Audio recorded standard transplant nurse education | 2.16 minutes educational video animation about high kidney donor profile index (KDPI) followed by a 2.05-minute animation about increased risk donor (IRD) in addition to audio recorded standard transplant nurse education  Watched once  <https://www.youtube.com/channel/UC3xXkG9VO83Bkj-jS09Vd3Q/videos> | **Patient knowledge mean:**  I: 7.4  C: 6.6  I vs C: p=0.036 (β = 0.23; 95% CI, 0.1-1.8) | **Patient Decisional Self-efficacy, mean (SD)**:  I: +1.6 (1.0)  C: +1.2 (1.2)  I vs C: p=NS | **Patient IRD Willingness mean (SD):** I: 0.7 (1.0) C: 0.05 (1.0),  I vs C: p=0.003  **Patient KDPI Willingness or Consent (Willingness to accept a KDPI >85%) mean (SD):** −0.5 (1.3) vs −0.5 (1.3), p=NS  **Proportion of subjects signing consent to receive KDPI >85% kidney**:  I: 27.5%  C: 13.5%,  I vs C: p=NS | **Knowledge:**  **Favours animation**  **Attitudes & cognitions:**  No difference  **Behaviours**:  Favours animation for IRD willingness only. | |
| Li 2019, China {Li, 2021 #217} | RCT | Participants with lung cancer, thoracic surgery department of a  large tertiary academic medical centre in Southern China  No statistical significant difference in education level | Age: 53 (9.2),  Male: 59% | N=80  I= 40  Animation education  C=40  Traditional face-to-face education | Standard animation intervention consisted of 3 sections totalling 31 minutes. These sections  Included a 6-minute animation introduction, a 10-minute nurse demonstration, and a 15-minute patient teach-back demonstration. animation  Watched twice each day at the patients’ bedside  Link NR | **Change in score of training related knowledge (OR, 95%CI):**  OR = 3.22, (95%CI 1.23 to 8.40), p=0.017 |  | **Change in score of exercise compliance** (**OR, 95%CI):**  OR = 1.42, (95%CI 0.56 to 3.64), p=NS | **Knowledge:**  Favours animation  **Behaviours:**  No difference between arms | |
| Mofrad 2021, Netherlands {Babapour Mofrad, 2021 #229} | RCT (3 arms) | Patients visiting the memory clinic of the Alzheimer Center Amsterdam  No statistical significant difference in education level between the 3 groups | Age 63 (9),  Male: I1: 53%, I2: 69%, C: 70% | N=209  I1=63  Animation viewing at home + Usual care  I2 =70  Animation viewing in clinic + Usual care  C=76  Usual care | 3 minutes animation video to inform and prepare patients and caregivers for the Lumber puncture (LP) procedure in the context of Alzheimer’s Disease (AD) diagnosis which included the LP procedure and the most common complications.  All participants also received care as usual.  Home viewing group were allowed to watch the video as often as desired. Clinic viewing group viewed the video once in the waiting room  Link NR | **Information recall:**  T2 (screening day)  I1 vs C :  mean difference 0.97(0.29) (p=0.003)  I2 vs C:  mean difference 1.35(0.28)(p<0.001)  T3 (end of screening day)  I1 vs C :  mean difference 0.84(0.26) (p=0.005)  I2 vs C:  mean difference 0.9(0.26)(p=0.002)  .  There was no difference in information recall between home and clinic viewing at either T2 or T3 | **Satisfaction:** no difference in satisfaction between interventions and control in terms of satisfactory F[2,176] = 2.24,  (p=NS) |  | **Knowledge**:  Favours animation  **Attitudes & cognitions**  No difference in satisfaction | |
| Saengow, 2018, Thailand {Saengow, 2018 #1375} | RCT | Paediatric patients who were diagnosed with epilepsy who have been visiting to routine service paediatric neurology clinic at Maharat Nakhon Ratchasima Hospital, Thailand  Education level NR | Age: I= 7.6 (4.5), C= 7.6 (4.8),  Male: 58% | N= 214  I=126  Video + advice  C=88  Advice only | 3 minutes animated video about appropriate use of anti-epileptics  Parents watched the video once via a portable DVD player  <https://www.youtube.com/watch?v=uEHmvl9qZlc> | Knowledge, mean change in score pre-post immediately post-intervention:  I: +0.5  C: +0.1  I vs C: p<0.001  Knowledge, mean change in score pre-post 3 months post-intervention:  I: +0.6  C: +0  I vs C: p<0.001 | Not assessed | Change in drug adherence:  I: 42.9%  C: 15.9%  I vs C: p<0.001 | | **Knowledge: favours animation.**  **Behaviour: favours animation.** |
| Wonggom 2020, Australia {Wonggom, 2020 #6337} | RCT | Heart failure patients, 3 outpatient clinics in three public hospitals in South Australia.  No statistical significant difference in education level | Age: 67.5 (11.3),  Male: 81% | N= 36  I= 17  Avatar app + usual care  C= 19  Usual care | Avatar app which is based on the Heart Foundation of Australia's booklet, ‘Living Well with Heart Failure’  Watched on tablet computer. Patients could watch as often, or as little as they would like during the clinic appointment  Link NR | Knowledge of heart failure (%), change in score pre-post at 30 days post-Int:  I: +15.3%  C: +3.5%  P=NS  Knowledge of heart failure (%), change in score pre-post at 90 days post-Int:  I: +22.2%  C: +3.7%  P=0.002 | Self-care confidence, change in score pre-post at 30 days post-Int:  I: +7.4%  C: +4.9%  P=NS  Self-care confidence, change in score pre-post at 90 days post-Int:  I: +19.6%  C: +5.0%  P=NS | Self-care behaviours, change in score pre-post at 30 days post-Int:  I: +16.4%  C: +7.0%  P=NS    Self-care behaviours, change in score pre-post at 90 days post-Int:  I: +11.0%  C: +6.8%  P=NS | | **Knowledge:** favours animation at 90 days; no difference between arms at 30 days.  **Attitudes & Cognitions:** no difference between arms.  **Behaviours:** no difference between arms. |

### Table 2: Category 3 -Topics related to Public Health, health promotion or illness prevention

| **Authors, years, country** | **Study design** | **Participants, Setting, Education level between the 2 groups** | **Age Mean (SD)/ % Male** | **Total sample;**  **Intervention descriptor & sample size (I);**  **Control descriptor & sample size (C)** | **Intervention details and link(if provided)** | **Results (Intervention vs Control)** (**Knowledge = knowledge or understanding. Attitudes & Cognitions = satisfaction or self-efficacy or confidence in decision, etc. Behaviours = behaviours or skills or intended behaviours) , p value**  | | | **Group favoured** |
| --- | --- | --- | --- | --- | --- | --- | --- | --- | --- |
|  |  |  |  |  |  | **Knowledge** | **Attitudes & Cognitions** | **Behaviours** |  |
| Bukkhunthod 2020, Thailand {Bukkhunthod, 2020 #299} | Cluster RCT (by class) | 9-12 year olds at school  Similar education level, school children | Age: I= 11.0, C=10.9,  Male: 50% | N=80  I=40  Animated educational intervention  C=40  Traditional programs, including pamphlets, posters, and educators | 1.43 minutes video animation about carcinogenic  liver fluke  watched 3 times in class  <https://www.youtube.com/watch?v=e3WEZ_n81K4> | **Knowledge, means score out of 10 (SD):**  I: 8.6 (0.9)  C: 4.7 (0.6)  I vs C: p=0.01  The proportion of pupils getting answer correct was greater in the animation group on all 10 quiz questions. |  |  | **Knowledge:**  Favours animation. |
| Burapasikarin 2020, Thailand {Burapasikarin, 2020 #6331} | RCT | Postpartum women who attended post-partum unit at the university hospital  No statistical significant difference in education level | Age: I= 29.8 (5.2); C= 29.9 (5.6),  Male: 0% | N=270  I=135  animated education video + standard care  C=135  Standard care | 7 minutes Long-acting reversible contraception (LARC) method animated educational video focusing on Implanon NXT®,Jadelle and copper intrauterine contraceptive  devices (IUD)  Watched once on a tablet computer  Link NR |  |  | **LARC utilization at 6–8 weeks n/N(%):** 78/135 (57.8%) (95% CI 49.0–66.2) vs 35/135 (25.9%)  (95% CI 18.8–34.2), (p<0.05) | **Behaviours**: favours animation |
| Choa 2008, Korea {Choa, 2008 #6332} | Cluster RCT | Hospital employees from the healthcare  Similar education level, no p values | Age: I=28.1 (NR) C= 28.4 (NR);  Male: I= 45.4%, C= 48.7% | N=85  I= 44  Animation-assisted (Cardiopulmonary resuscitation)CPR  C= 41  Dispatcher-assisted CPR | CPR animation based CPR self-training tool for a lay person. The length of the animation is not reported.  Watched on mobile phone, frequency NR  Link NR | NR | NR | CPR Airway management, assessed mean score out of 4 (SD):  I: 3.4 (0.1)  C: 3.1 (0.1)  P=0.04  CPR Rescue breathing, assessed mean score out of 12 (SD):  I: 8.3 (0.2)  C: 7.1 (0.2)  P=0.003  CPR Chest compression, assessed mean score out of 12 (SD):  I: 8.9 (0.4)  C: 6.6 (0.4)  P=0.002  Ventilation (% skills rated as adequate by ResusciAnne. SD).  Adequate volume:  I: 14.1% (2.6)  C: 14.2% (2.7)  P=NS  Adequate flow rate:  I: 22.8% (5.6)  C: 26.8 (5.8)  P=NS  Compression (% skills rated as adequate by ResusciAnne. SD).  Correct position:  I: 68.8% (3.6)  C: 56.5% (3.7)  P=0.033  Adequate rate:  I: 72.4% (3.7)  C: 57.6% (3.8)  P=0.015  Adequate depth:  I: 30.0% (4.7)  C: 24.2% (NR)  P=NS  Time taken to give first breath, seconds:  I: 63.7 (1.4)  C: 79.4 (1.4  P<0.001  Time taken to give first chest compression, seconds:  I: 98.7 (1.9)  C: 138.2 (1.9)  P<0.001  Time taken to complete first CPR cycle, seconds:  I: 117.6 (2.0)  C: 161.7 (2.2)  P<0.001 | **Behaviours:**  Favour animation in checklist assessment and time interval compliance but no difference in psychomotor skill measures |
| Housten, 2020, USA {Housten, 2020 #307} | RCT (3 Arms) | Participants who came to a community food bank or the Houston (Texas) Cancer Prevention centre  Similar education level, no p values | Age(range): 45 to 75  Male: 37%. | N= 187  I= 63  Video with animated images  C1= 62  Video with static images  C2= 62  Audio booklet | 3.3 minutes of the elements of risk communication relevant to colorectal cancer (CRC) screening  Watched once  Link provided by the authors (confidential) | Knowledge, total % mean score (SD):  I: 65.0 (18.6)  C1: 62.0 (16.4)  C2: 64.6 (18.5)  I vs C2: p=NS  I vs C1: NR  Verbatim knowledge, mean % score (SD):  I: 56.6 (23.2)  C1: 55.0 (23.7)  C2: 58.3 (21.4)  I vs C2: p=NS  I vs C1: NR  Gist knowledge, mean % score (SD):  I: 76.2 (23.4)  C1: 71.2 (23.4)  C2: 78.2 (16.7)  I vs C2: p=NS  I vs C1: NR | NR | Not assessed | **Knowledge:**  No difference between arms |
| Leiner 2004, USA {Leiner, 2004 #959} | RCT | Patients & parents receiving polio vaccine in Paediatric Clinic  No statistical significant difference in education level | NR | N=192  I= 96  Animated cartoon video  C= 96  Reading printed information sheet | Approximately 8 minutes animated cartoon video which portrayed a mother taking her baby to the pediatrician to receive the polio vaccine and emphasize how there is actually very little discomfort. Also the information about the vaccine, risks and contact information in case of an adverse effect.  Animation frequency and delivery NR  Link NR | **Knowledge, mean score out of 8 (SD):**  I: 6.2 (1.7)  C: 5.0 (1.9)  I vs C: p<0.001 | n/a | n/a | **Knowledge:** Favours animation |
| Meppelink 2015, Netherlands {Meppelink, 2015 #2622} | RCT | Participants age 55 years or older with either low or high health literacy  No difference in education, no p values | Age: 68.2 (8.6),  Male:52.4% | N= 231  I= 117  Animation  C=114  Static illustration  (participants also randomly allocated to receive either spoken or written information) | The animation which included the messages about colorectal cancer screening (i.e. the development of the disease, why early detection is beneficial, the procedure of the test and the possible test outcomes)  Frequency and delivery NR  [https://www.ncbi.nlm.nih.gov/pmc/articles/PMC4319081/#app1](https://www.ncbi.nlm.nih.gov/pmc/articles/PMC4319081/%23app1) | Knowledge, mean score out of 28 (95% CI):  Participants with low health literacy:  Written information:  I: 8.7 (6.9-10.4)  C: 9.6 (7.7-11.5)  P=NS  Spoken information:  I: 13.2 (11.0-15.5)  C: 9.6 (7.5-11.7)  P=0.02  Participants with high health literacy:  Written information:  I: 15.1 (13.2-17.0)  C: 14.5 (12.7-16.3)  P=NS  Spoken information:  I: 15.5 (13.7-17.3)  C: 16.0 (14.1-17.9)  P=NS | NR | Not assessed | **Knowledge:**  Favours animation in the low health literacy when the animation combined with spoken text (1 out of 4 groups). No difference between arms in the high health literacy groups and the low health literacy, written animation group ( 3 out of 4 groups) |
| Rakhmilla 2018, Indonesia {Rakhmilla, 2018 #1384} | Quasi-experimental design (3 Arms) | Students of Senior High School in Jatinangor sub-district in Indonesia  Similar education level, all in senior high school | Age: Conventional group: <16 yrs: 37 (68.5%) >16 yrs: 17 (31.5%), video animation group: <16 yrs: 40 (93%), >16 yrs: 3 (7%), Peer group: <16 yrs, 56 (93.3%), >16 yrs: 4 (6.7%)  Male: 41% | N= 180  I=60  Video animation  C1= 60  Peer education  C2=60  Conventional lecture education | 9 minutes video animation consisting of pictures and writing on thalassemia prevention  Delivered in a classroom setting, frequency NR  Link NR | Knowledge, mean score (SD):  I: 14.9 (1.5)  C1: 14.9 (2.0)  C2: 11.8 (2.5)  I vs C1: NR  I vs C2: p<0.001 | Not assessed | Not assessed | **Knowledge:** favours animation |
| Romantika, 2020, Indonesia{Romantika, 2020 #251} | Quasi RCT | Mothers who have children aged 4-7 years in Indonesia  Mother participants had either high school education or less, difference between the groups was NR | Age (Mothers age range): 17 to 55 yrs  Male: 0% | N= 120  I= 71  2D animated video  C= 49  Leaflets | 6 minute 2D animated video which discussed definitions of behavioural problems, risk factors, the impact of behavioural problems.  Watched once  Link NR | Knowledge, change scores, median (min-max):  I: +3 (-4, 11)  C: +2 (-2, 6)  I vs C: p=0.004 | Attitude to information, change scores, median (min-max):  I: +3 (-3, 20)  C: +2 (-17, 9)  I vs C: p<0.046 |  | **Knowledge**: favours animation**.**  **Attitudes & Cognitions:** attitudes favours animation**.** |
| Ruparel, 2019, United Kingdom {Ruparel, 2019 #314} | RCT | Smokers/former smokers participants were invited to a London hospital (UCL or Homerton University Hospital) for a lung health check (LHC)  Participants had either Bachelor degree or less, differences between the groups was NR | Age (range): 60 to 76 yrs  Male: 48% | N= 246  I= 126  Animation film + booklet  C= 120  Booklet alone | 5.30 minutes information film which discussed lung cancer, the benefits and harm of lung cancer screening, the low-dose CT procedure and the possible results after the scan  Participants were allowed 10 minutes to watch the film and/or read the booklet  [AnnalsATS.201811-841OC_ucl_lung_cancer_animation_v2_05_08_2016.mp4](https://www.ncbi.nlm.nih.gov/pmc/articles/PMC6543473/bin/AnnalsATS.201811-841OC_ucl_lung_cancer_animation_v2_05_08_2016.mp4) | Knowledge, change in median score out of 10 pre-post:  I: +3  C: +2  I vs C: p<0.001  Knowledge, change in mean score out of 10 pre-post (SD):  I: 2.2 (1.8)  C: 1.8 (1.9)  I vs C: p<0.001 | ‘Subjective knowledge’, change in mean score out of 5 pre-post (SD):  I: 0.9 (1.0)  C: 0.5 (1.1)  I vs C: p<.02 (after adjustment for baseline variables)  Decisional conflict (certainty), mean score (SD):  I: 8.5 (1.3)  C: 8.2 (1.5)  I vs C: p=0.007 | NR | **Knowledge:**  Favours animation.  **Attitudes & Cognitions:**  ‘Subjective knowledge’ favours animation.  Decisional certainty: favours animation. |
| Schnellinger, 2010, USA {Schnellinger, 2010 #819} | RCT | Parents of the paediatrics patients in an urban paediatric emergency department  No statistical significant difference in education level | Age median (range): 16 to 72  Male: NR | N= 162  I =83  Animated video  C1 =79  Pamphlet  C2= no intervention (therefore not included in the SR data set) | 3 minute animated video about appropriate use of antibiotics  Parents watched the video once via a portable DVD player  Link NR | Knowledge, score, mean rank, 1-2 hours post-intervention:  I: 149.3  C1: 136.7  I vs C1: p=0.19  Knowledge, score, mean rank, 4 weeks post-intervention:  I: 111.8  C1: 94.3  I vs C: p=0.04  Change in knowledge score pre-post at 1-2 hours post-intervention:  I: increase p<.001  C1: increase p<.001  Change in knowledge score pre-post at 4 weeks post-intervention:  I: increase p<.001  C1: increase p<.001 | Evaluation items, mean scores:  Interesting or useful:  I: 92.8  C1: 94.9  I vs C1: NS  Learnt something:  I: 72.3  C1: 83.5  I vs C1: NS | Not assessed | **Knowledge:** favours animation at 4 weeks but not immediately after post intervention (1-2 hrs)  **Attitudes & Cognitions:** no difference between arms |
